# Efficacy, drug sustainability, and safety of ustekinumab treatment in Crohn’s disease patients over three years

**DOI:** 10.1038/s41598-024-65987-1

**Published:** 2024-06-28

**Authors:** Laszlo J. Barkai, Lorant Gonczi, Fruzsina Balogh, Dorottya Angyal, Klaudia Farkas, Bernadett Farkas, Tamas Molnar, Tamas Szamosi, Eszter Schafer, Petra A. Golovics, Mark Juhasz, Arpad Patai, Aron Vincze, Patricia Sarlos, Alexandra Farkas, Zsolt Dubravcsik, Tamas G. Toth, Hajnal Szekely, Pal Miheller, Peter L. Lakatos, Akos Ilias

**Affiliations:** 1 Department of Internal Medicine and Oncology, Semmelweis University, Koranyi Sandor Utca 2A, 1083 Budapest, Hungary; 2 Department of Medicine, University of Szeged, Szeged, Hungary; 3 Department of Gastroenterology, Medical Centre, Hungarian Defence Forces, Budapest, Hungary; 4 Department of Medicine, St. Margit Hospital, Budapest, Hungary; 5 Department of Medicine and Gastroenterology, Markusovszky Hospital, Szombathely, Hungary; 6 First Department of Medicine, Medical School, University of Pecs, Pecs, Hungary; 7 Department of Gastroenterology, Bács-Kiskun County Hospital, Kecskemet, Hungary; 8 Department of Gastroenterology, St. Janos Hospital, Budapest, Hungary; 9 Department of Surgery, Transplantation and Gastroenterology, Semmelweis University, Budapest, Hungary; 10 Montreal General Hospital, McGill University Health Center, Montreal, Canada

**Keywords:** Crohn’s disease, Biological therapy, Biologicals, Inflammatory bowel disease, Ustekinumab, Long-term, Inflammatory bowel disease, Crohn's disease

## Abstract

Long-term data on ustekinumab in real-life Crohn’s disease patients are still missing, though randomized controlled trials demonstrated it as a favorable therapeutic option. We aimed to evaluate ustekinumab's clinical efficacy, drug sustainability, and safety in a prospective, nationwide, multicenter Crohn’s disease patient cohort with a three-year follow-up. Crohn’s disease patients on ustekinumab treatment were consecutively enrolled from 9 Hungarian Inflammatory Bowel Disease centers between January 2019 and May 2020. Patient and disease characteristics, treatment history, clinical disease activity (Harvey Bradshaw Index (HBI)), biomarkers, and endoscopic activity (Simple Endoscopic Score for Crohn’s Disease (SES-CD)) were collected for three-years’ time. A total of 148 patients were included with an overall 48.9% of complex behavior of the Crohn’s disease and 97.2% of previous anti-TNF exposure. The pre-induction remission rates were 12.2% (HBI), and 5.1% (SES-CD). Clinical remission rates (HBI) were 52.2%, 55.6%, and 50.9%, whereas criteria of an endoscopic remission were fulfilled in 14.3%, 27.5%, and 35.3% of the subjects at the end of the first, second, and third year, respectively. Dose intensification was high with 84.0% of the patients on an 8-weekly and 29.9% on a 4-weekly regimen at the end of year 3. Drug sustainability was 76.9% during the follow-up period with no serious adverse events observed. Ustekinumab in the long-term is an effective, sustainable, and safe therapeutic option for Crohn’s disease patients with severe disease phenotype and high previous anti-TNF biological failure, requiring frequent dose intensifications.

## Introduction

Crohn’s disease (CD) is a progressive and destructive inflammatory bowel disease (IBD) with an overall unpredictable nature^1^. Therapeutic options for CD have extensively improved since the introduction of biological agents with an anti-TNF or a selective anti-integrin effect. While the appropriate prognostic markers for primary and secondary non-response to these treatments are still awaited, a large proportion of patients might require novel therapeutic options with a target against different inflammatory pathways^2^. Ustekinumab is a fully human, immunoglobulin G1 monoclonal antibody. It targets the standard p40 subunit of the IL-12 and IL-23 cytokines preventing their interactions with the IL-12 and IL-23 receptors of T cells, antigen-presenting cells, and natural killer cells. This results in reduced activity via negative feedback on chronic immunological responses of CD^3^.

The UNITI-1 and UNITI-2 were phase 3 randomized controlled trials (RCTs) of 8-week induction therapy with ustekinumab (UST) in moderate-severe CD, where patients were exposed to or naïve to anti-TNF therapy. The IM-UNITI was their 44-week maintenance trial, and its long-term extension (LTE) was continued until five years’ time follow-up. UST was demonstrated as a safe therapy with good efficacy in inducing and maintaining clinical and endoscopic remission in these RCTs^4,5^. Though RCTs provide satisfactory treatment efficacy and safety information in ideal conditions, real-life studies of routine circumstances and an unselected patient population can capture more detailed treatment characteristics.

UST was approved by both the US Food and Drug Administration and the European Medicines Agency for the treatment of CD in 2016, and it was adopted for general reimbursement by the National Health Insurance Fund of Hungary (NEAK) in October 2018.

Previously, we published results of favorable drug sustainability and clinical efficacy of 1-year UST treatment in a Hungarian multicenter CD patient population with severe disease phenotype and previous anti-TNF failure^6^.

The present study aimed to evaluate the three-year treatment efficacy (clinical and endoscopic remission rates, UST dose intensification), drug sustainability and safety of UST in CD patients based on a multicenter prospective cohort from Hungary.

## Methods

### Study design and patients

This is a prospective multicenter observational study conducted at 9 Hungarian tertiary referral IBD centers (4 academic and 5 regional centers). Between January 2019 and May 2020, patients older than 18 years receiving UST as a treatment for their CD were consecutively enrolled. Follow-up was conducted until November 2022.

A standardized monitoring strategy was applied in all participating centers, as the Hungarian National Health Fund requested. Data on patient demographics, disease phenotype, and treatment history (surgical history, previous and present concomitant medications) were collected from the electronic medical records and upon patient inclusion. Disease characteristics were assessed according to the Montreal classification^7^.

Patients for induction treatment were given a single dose of UST intravenous injection using a weight-based dosage regimen: 260 mg < 55 kg, 390 mg between 55 and 85 kg, and 520 mg > 85 kg. This was followed by subcutaneous (s.c.) injections (90 mg) starting at week 8 (w8) and was continued with s.c. injections every 12 weeks, constituting the maintenance treatment. In the case of dose-intensification, s.c. injections were given every 8 (Q8 regimen) or every 4 weeks (Q4 regimen).

The primary outcomes of the present study were the evaluation of clinical remission and dose intensification rates, along with the sustainability and safety of UST. The secondary outcome was the endoscopic remission rate throughout a three-year follow-up period. Clinical and endoscopic disease activities and data collection on biomarkers were performed at baseline, 1st-year, 2nd-year, and 3rd-year follow-up visits. To obtain a thorough and sensitive analysis of our data, we chose to simultaneously evaluate the two most commonly used clinical disease activity scores in CD: the Harvey Bradshaw Index (HBI) and the Crohn’s Disease Activity Index (CDAI)^8,9^. The endoscopic activity was quantified using the Simple Endoscopic Score for Crohn’s Disease (SES-CD)^10^. Infusion and injection-related adverse events were registered at baseline and every follow-up visit.

Clinical disease activities were defined as moderate-to-severe (HBI > 7), mild (5 ≤ HBI ≤ 7), or within the range of clinical remission (HBI < 5), based on these ranges. SES-CD scores of 3–6, 7–15, and > 15 indicate mild, moderate, or severe endoscopic activity, respectively. In fistulizing disease, remission was defined as no fistula drainage on gentle pressure during the physical exam. Biomarker evaluation consisted of C-reactive protein (CRP) measurement. The cut-off level for CRP was set at < 10 mg/L. We calculated the ‘composite clinical and biomarker remission’ rates (composite remission), defined by the coexistence of HBI scores within the range of clinical remission and CRP levels below cut-off (HBI < 5 and CRP < 10 mg/L). Data on concomitant systemic therapy with corticosteroids was collected.

### Statistical analysis

Descriptive statistics were applied to characterize demographic data, remission rates, and adverse events. Medians and interquartile ranges (IQR) were calculated for continuous variables. Kaplan–Meier survival curves were used to evaluate drug sustainability and dose intensification. Statistical analysis was performed using SPSS software v.20.0 (Chicago, IL); p < 0.05 was considered statistically significant.

### Ethics declaration

This study was performed in line with the principles of the Declaration of Helsinki. Ethical approval was acquired from the Hungarian Medical Research Council’s Committee of Scientific and Research Ethics [ETT-TUKEB 20,877–1/2019/EKU].

### Consent to participate

Informed Consent forms of all patients were obtained following the Helsinki Declaration.

## Results

### Initial clinical characteristics

A total of 148 patients were included in the study and were followed up for a median time of 166 weeks (IQR 144–186). Detailed patient characteristics are summarized in Table 1. The median age at inclusion was 35 years, the disease duration was 15 years, and 59.5% of the subjects were female. As per the age of diagnosis of the CD, 70.8% fell into the A2 category of the Montreal classification. Both ileal and colonic involvement (Montreal L3) were present in 55.9% of the subjects. Complex behavior of the CD was observed in 48.9% (Montreal B2 + B3) with perianal manifestations in 45.6% of the subjects. Prior surgical resection and perianal intervention rates were 46.3% and 46.2%, respectively. The previous anti-TNF exposure was 97.2%, while the previous vedolizumab failure was 25.9%.

**Table 1:** Initial patient characteristics.

Gender (male/female; n)	60/88
Age at inclusion (median (IQR); years)	35 (28–45)
Disease duration (median (IQR); years)	15 (10–20)
Smoking (No, Yes, Stopped; %)	75.2/17.8 / 7
Age at CD diagnosis (Montreal A1 / A2 / A3; %)	17.5/70.8 / 11.7
Location (Montreal L1 / L2 / L3 / + L4; %)	16.9/22 / 55.9 / 5.2
Behavior (Montreal B1 / B2 / B3; %)	51.1/26.3 / 22.6
Perianal manifestation (%)	45.6
Previous resective surgery (%)	46.3
Previous perianal surgery (%)	46.2
Previous immunosuppressive therapy (%)	82.4
Previous anti-TNF therapy (None / IFX / ADA / IFX + ADA; %)	2.8/14.2/19.9/63.1
Previous vedolizumab therapy (%)	25.9
Follow-up time (median (IQR); weeks)	166 (144–186)

*CD* Crohn’s disease, *IQR* interquartile range, *anti-TNF* anti-tumor necrosis factor, *IFX* infliximab, *ADA* adalimumab.

At the start of the study, subjects all over showed moderate-to-severe disease activity with median (IQR) HBI, CDAI, and SES-CD scores of 10.0 (6–15), 292.0 (200–323), and 14.5 (8–18), respectively. Mild clinical activity was registered in 17.6% (5 ≤ HBI ≤ 7), and mild endoscopic activity was present in 9.4% (SES-CD: 3–6) of the cases. Clinical remission rates were 12.2% based on HBI < 5, composite remission rate was 9.5%, whereas 5.1% were in an endoscopic remission (SES-CD < 3). Of note, parallel systemic corticosteroid medication was present in as high as 32.4% of the patients at baseline.

### Remission rates on follow-up

**Figure 1:**
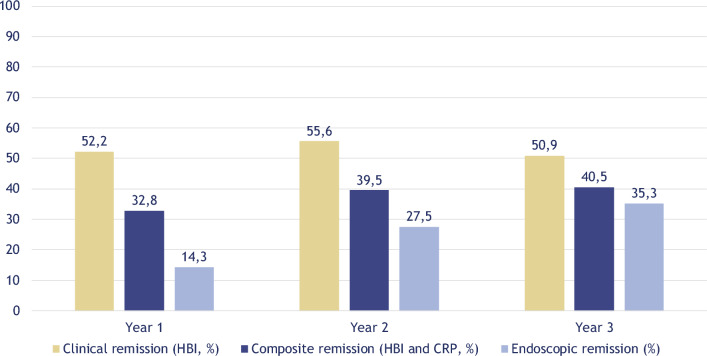
Remission rates during follow-up. Clinical remission was defined by a Harvey Bradshaw Index (HBI) < 5, composite remission upon HBI < 5 and CRP < 10 mg/L, and endoscopic remission upon the Simple Endoscopic Score for Crohn’s Disease (SES-CD) < 3.

Clinical remission rates did not show any significant change throughout the first, second, and third-year follow-up with values of 52.2%, 55.6%, and 50.9%, respectively (Fig. 1). Of note, clinical remission rates computed with CDAI were 63.4%, 65.5%, 64.5%, respectively. When computing composite clinical and biomarker remission, rates were at 32.8%, 39.5%, and 40.5%, respectively. The median (IQR) endoscopic disease activity (SES-CD) values were 9.0 (5.5–12), 8.5 (2–14), and 4.5 (2–10.8), whereas 14.3%, 27.5%, and 35.3% of the subjects were in an endoscopic remission at the end of the first, second, and third year of the study, respectively (Fig. 1). It has to be noted, that follow-up endoscopy results were only available for 28 (18.9%), 40 (27.0%), and 34 (23.0%) of the 148 patients within the first, second, and third-year follow-up, respectively. Rates of concomitant systemic therapy with corticosteroids among the cohort were 12.8%, 7.4%, and 5.4% by the end of the first, second, and third years.

### Dose intensification, drug sustainability and safety

**Figure 2:**
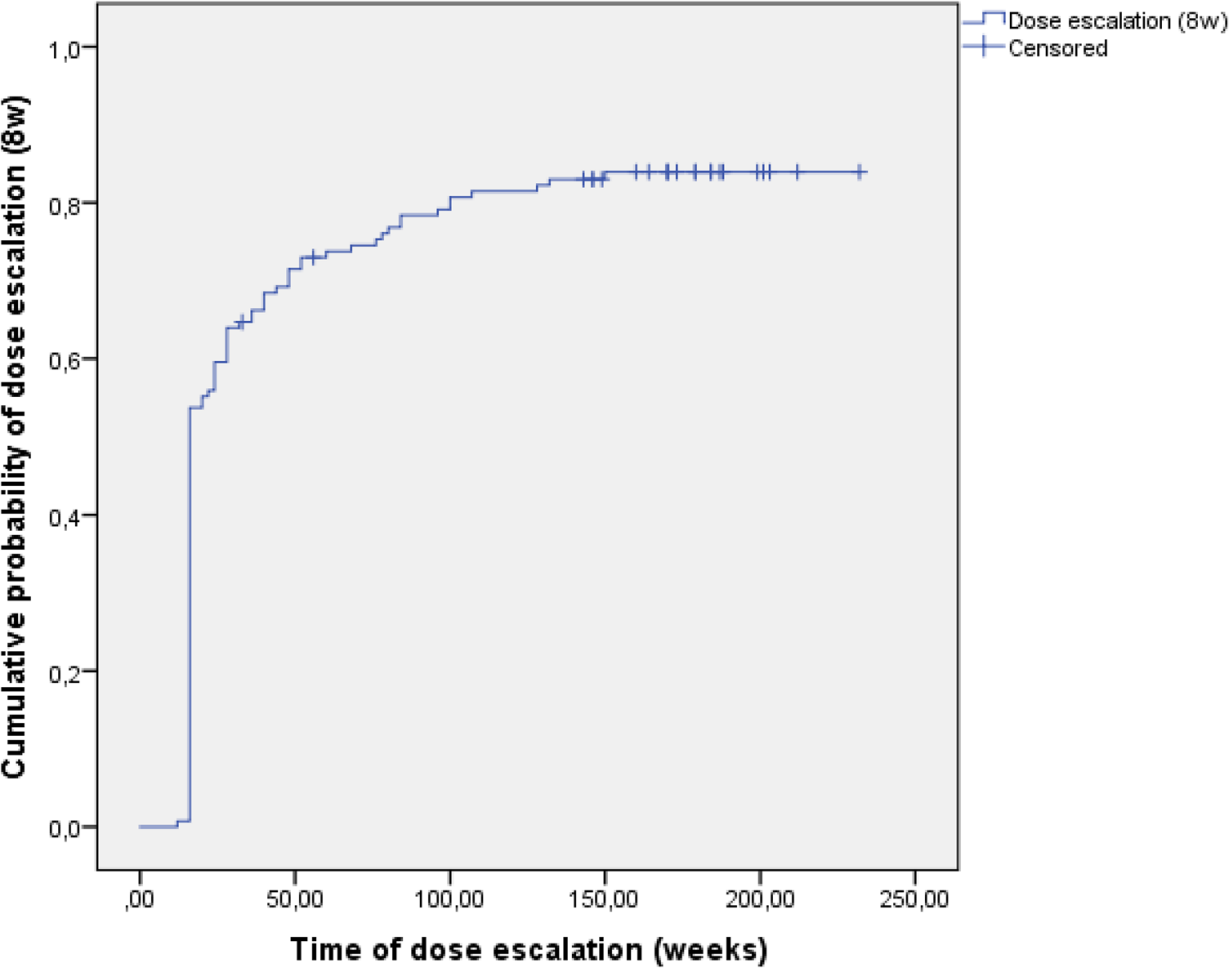
Probability of dose intensification to an 8-weekly (8w) regimen in Kaplan–Meier analysis.

**Figure 3:**
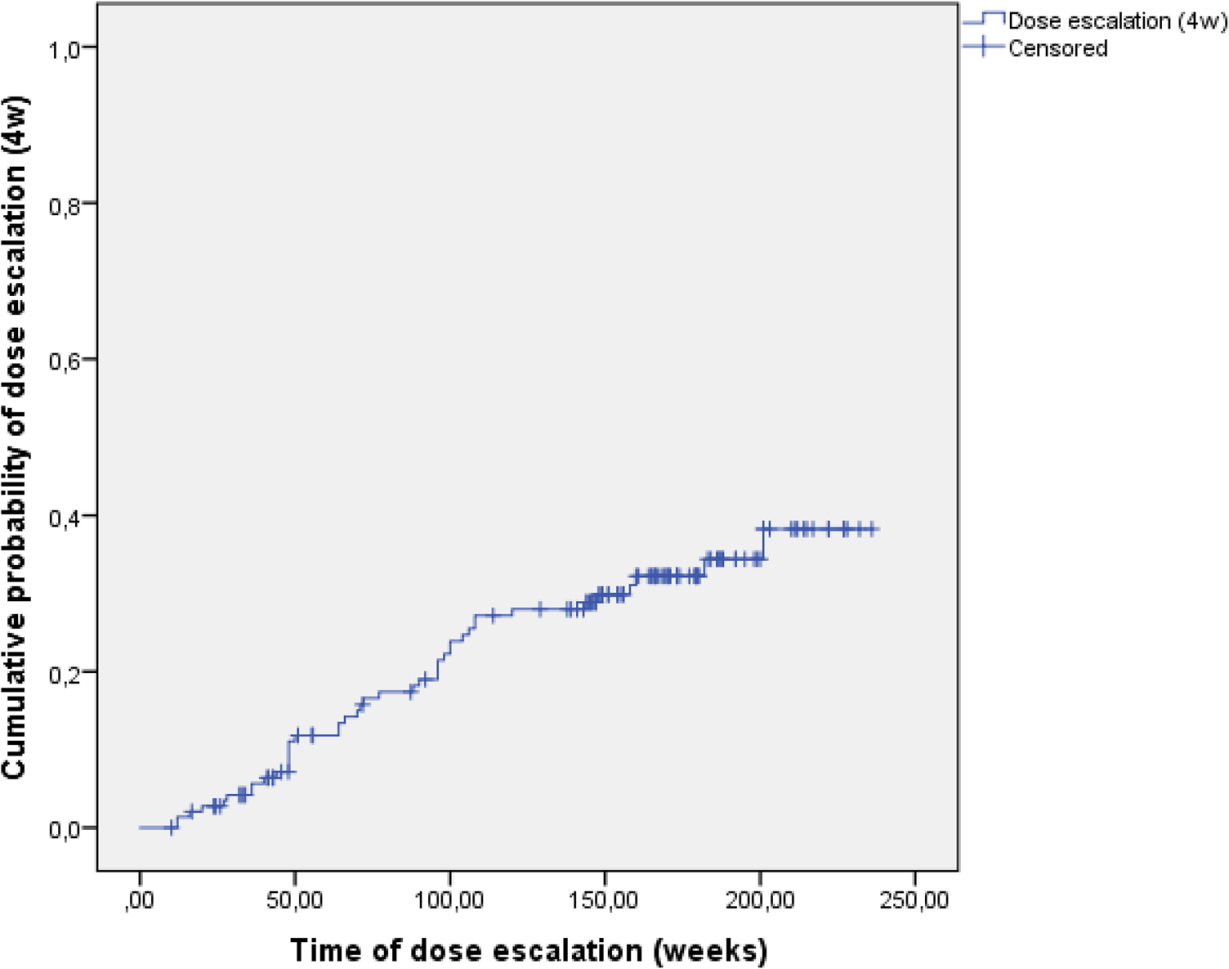
Probability of dose intensification to a 4-weekly (4w)regimen in Kaplan–Meier analysis.

Intensifying the dose of UST to a Q8 regimen occurred frequently and at an early stage of the treatment: 73.0% (SD: 3.8) of the subjects at year 1 and 84.0% (SD: 3.2) at year 3 (Fig. 2). These numbers involve subjects who later needed further dose intensification to a Q4 regimen. Q4 regimen were given to 11.8% (SD: 2.8) of all patients at year 1 and 29.9% (SD: 4.1) at year 3 (Fig. 3).

**Figure 4:**
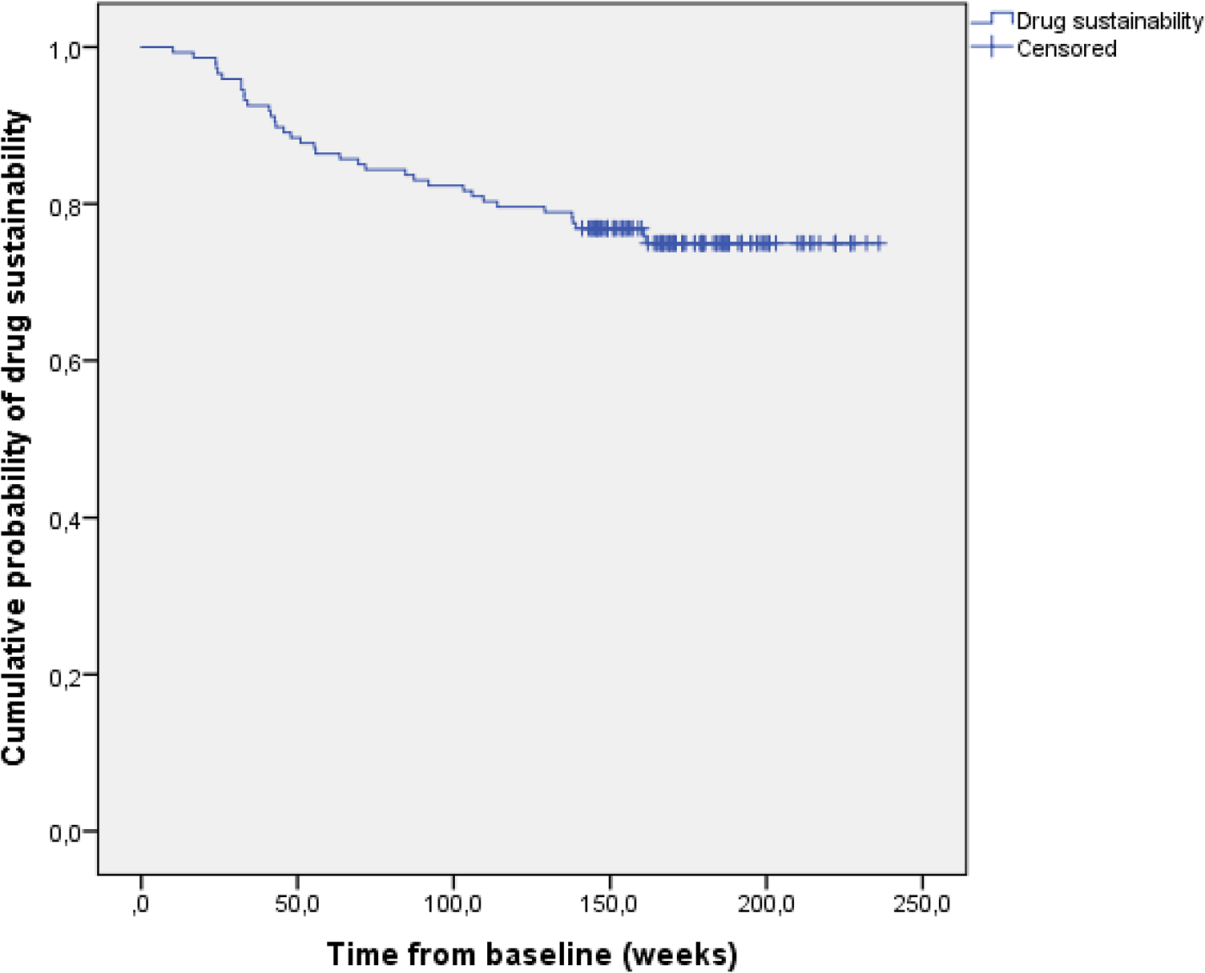
Drug sustainability in Kaplan–Meier analysis.

Drug sustainability was high, with 76.9% (SD: 3.5) of patients remaining on therapy at year three (Fig. 4). Overall 36 patients discontinued therapy during our follow-up with the following reasons: loss of response: n = 24; surgical intervention: n = 6; loss to follow-up: n = 3; other n = 2 (pregnancy, and initiation of tofacitinib due to a rheumatological indication); side effect n = 1 (new development of cystic non-malignant hepatic lesions of an unknown origin). No serious adverse events were observed in our cohort.

## Discussion

The results of our multicenter prospective study provide evidence of the long-term efficacy, drug sustainability and safety of UST treatment in an unselected real-world CD patient cohort with complex disease phenotype and a high rate of previous anti-TNF failure. Remission rates were maintained throughout the 166 weeks of follow-up time. The overall probability of dose-intensification was high (84.0%), and the majority of our subjects (76.9%) remained on therapy by the end of year 3 with no medication-related serious adverse events experienced.

At the end of years 1, 2, and 3, 52.2%, 55.6%, and 50.9% of the patients were in a clinical remission as per HBI, and 63.4%, 65.5%, and 64.5% as per CDAI, respectively. When assessing the composite clinical and biomarker remission (computed with HBI and CRP), 32.8%, 39.5%, and 40.5% of total subjects reached these criteria, respectively. In harmony with this, systemic corticosteroid therapy augmenting the UST treatment showed a decreasing trend with 12.8%, 7.4%, and 5.4%.

Data on long-term outcomes of UST treatment in CD patients is sparse especially the ones of real-world studies. In our previous publication, we showed favorable drug sustainability and clinical efficacy of this patient population throughout a 1-year follow-up^6^. The results were comparable to those of the RCTs^4,5^, and the previously published data from other unselected, mainly retrospective real-world studies^11–15^. The 2 years follow-up data of the IM-UNITI LTE trial found that 64.4% of all patients on an 8-weekly UST regimen (Q8) and 64.3% on a 12-weekly regimen (Q12) were in clinical remission based on CDAI at week 92^16^. At year 3, the clinical remission rate of the Q8 dosage patients was 69.5% whereas it was 61.9% for the Q12 regimen subjects^17^. These results are coherent with our data of the present study (remission rate of 55.6% in year 2 and 50.9% in year 3, based on HBI). The IM UNITI trial, due to its design, selectively involved CD patients only with a moderate-to-severe baseline clinical disease activity and a post-induction clinical response, whereas we enrolled subjects in a consecutive way. Yet, our baseline CDAI value of 292.0 can be matched with the ones of the IM UNITI cohort (320.4 in the Q12 and 313.1 in the Q8 treatment regime group) highlighting the strength of our results^4^.

Upon our thorough review of the literature, we found no available data with 3 years follow-up of UST-treated CD patients of real-world settings. The few 2-year studies concluded with all-over similar findings to the results we publish here. Recent multicenter prospective data revealed clinical remission rates, as per the HBI, to be at 40.0% at year 1 and 40.4% at year 2, and steroid-free remission rates to be 35% and 35.5%, respectively^18^. The low but still present steroid use observed in our cohort (12.8%, 7.4%, and 5.4%) allows us to believe our steroid-free remission rates correspond to this degree (exact values for this variable of our cohort are not shown here).

A Swedish paper published HBI remission rates to be maintained at 32% and 29% at weeks 52 and 104, respectively^19^. Though these numbers might seem somewhat lower than our rates (52.2% and 55.6%), we have to note that 85% of their subjects continued treatment with the initial 12-week dosing interval by the end of year 2. In comparison, dose intensification within our cohort occurred in 73.0% by the end of the first and 84.0% of the third follow-up year which might be a reason for our better remission rates.

Our endoscopic remission rates (5.1% at baseline, 14.3%, 27.5%, and 35.3% at years 1, 2, and 3, respectively) and SES-CD values (14.5 at baseline, 9.0, 8.5, and 4.5 at years 1, 2, and 3, respectively) might also support the long-term potency of UST. Of note, a numerically improving trend can be identified throughout, however, in the context of the relatively low available data on follow-up endoscopy procedures in our cohort (18.9%, 27.0%, and 23.0%) we did not perform detailed statistical analyses on this.

Our findings on endoscopic remission at year 1 (14.3%) can be matched with the ones of the STARDUST trial which showed week 48 rates upon the treat-to-target and the standard-of-care treatment plans to be 11% and 15%, respectively. Only a few of the real-world studies focus on endoscopic improvement of UST treated CD patients, and even less contain precisely comparable results. A previous multicenter study observed an 82% endoscopic response to UST in CD patients within 26.6 months, however, it is hard to draw comparison to our data as their usual clinical and endoscopic scores are not shown. A different study found that 64.4% of their patients maintained endoscopic or radiographic response at week 52, yet details of pure endoscopic activity or remission are not available^21^. Moreover, both these were findings of retrospective studies. In 2018, Rutgeerts et al. demonstrated prospective data of a 2.5 points SES-CD decrease from baseline to week 44 in a UST-treated CD patient cohort with severe baseline endoscopic activity (15.4 for all patients), however, this change in the SES-CD values was not significant, and they did not perform any further follow-up of the cohort^5^. In a recent Finnish nationwide study CD patients on UST therapy were consecutively enrolled, and followed up for 14.2 months^22^. Complex disease phenotype with stricturing and penetrating behavior was comparable to our cohort (69 out of 155 patients = 44.5% versus 48.9%), however, only 64.9% of the subjects had a clinically active disease at baseline. SES-CD values decreased from 10 to 3 by the end of their 1st follow-up year, which fulfils the criteria of an endoscopic response. In comparison to our cohort, their baseline SES-CD values were lower (10 versus 14.5 in our cohort), indicating a less severe disease activity. This might explain the numerically mildly greater endoscopic response of the Finnish cohort in comparison to our subjects.

We detected high drug sustainability with more than three-quarters (76.9%) of the patients still on UST therapy by the end of the third year. The majority of the subjects went through and early dose intensification (73.0% at year 1 and 84.0% at year 3). The main reason for discontinuation was loss of response, and we did not find any serious adverse events during the follow-up period. Recent prospective data of the Dutch ICC (Initiative on Crohn and Colitis) Registry showed UST treatment probability to be at 64.3% at week 52 and 54.8% at week 104, with loss of response to be the main reason for quitting the drug, and no new safety issues^12^. A different Dutch study with partly the same authors as the ICC Registry study found a 59% drug survival probability at week 104^18^. The dose escalation rate was 33% at the end of year 1 and 15% at year 2. In a Brazilian prospective study, the UST discontinuation rate was 23% which is closer to our results of drug sustainability. Their treatment was also well tolerated during the 80 weeks of follow-up, though they mention a serious adverse event rate of 21% with mostly infectious causes^23^. Comparing these results of the different studies is challenging. Adverse events are not always defined in a homogenous way as seen in the Brazilian findings. Dose intensification rates are highly dependent not just on the disease severity of the cohorts in question, but also on the dynamically changing reimbursement regulations of different countries. A significant proportion of our subjects failed previous biologicals (both anti-TNFs and vedolizumab) leaving them in a position where UST was the last resort treatment option in Hungary at the time. Thus, some patients could have continued on UST (even on an increased dosage regimen) without strong, objective evidence of benefit on their disease. This could have affected our drug sustainability and even the dose intensification rates.

A limitation of our study is the lack of UST antidrug antibody (ADA) measurements. ADAs are linked with adverse events and frequent infusion reactions to biological treatments^24^. The formation of ADAs, in general, is thought to be an important factor in promoting loss of response to biological therapy. Patients treated with UST were reported to have lower rates of ADAs in comparison to infliximab, adalimumab or certolizumab^25^. Moreover, UST ADAs seem to rather not have an effect on treatment efficacy^17^. A previous meta-analysis found no significant difference in short-term AEs between patients treated with UST versus placebo^26^. These data from the literature are coherent with our observations on the effective maintenance of remission parallel to the low adverse event rates of UST throughout the 3 follow-up years.

The strengths of our study include a long-term prospective design of a large unselected patient group and a nationwide harmonized monitoring practice across all participating centers. To date, no real-world data of 3-year follow-up results of UST-treated CD patients are available in the literature.

In conclusion, UST showed favorable long-term clinical and endoscopic efficacy, and drug sustainability with no concerns about safety in a real-life multicenter cohort of multiple biological-exposed CD patients having high rates of complex disease phenotypes throughout a 3-year follow-up period.

## Data Availability

The data underlying this article will be shared on reasonable request by the corresponding author.
